# Transient transition from Stable to Dissipative Assemblies in Response to the Spatiotemporal Availability of a Chemical Fuel

**DOI:** 10.1002/anie.202414495

**Published:** 2024-11-11

**Authors:** Haridas Kar, Rui Chen, Krishnendu Das, Leonard J. Prins

**Affiliations:** ^1^ Department of Chemical Sciences University of Padua Via Marzolo 1 35131 Padua Italy

**Keywords:** systems chemistry, dissipative self-assembly, active matter, reaction-diffusion, hydrogel

## Abstract

The transition from inactive to active matter implies a transition from thermodynamically stable to energy‐dissipating structures. Here, we show how the spatiotemporal availability of a chemical fuel causes a thermodynamically stable self‐assembled structure to transiently pass to an energy‐dissipating state. The system relies on the local injection of a weak affinity phosphodiester substrate into an agarose hydrogel containing surfactant‐based structures templated by ATP. Injection of substrate leads to the inclusion of additional surfactant molecules in the assemblies leading to the formation of catalytic hotspots for substrate conversion. After the local disappearance of the substrate as a result of chemical conversion and diffusion the assemblies spontaneously return to the stable state, which can be reactivated upon the injection of a new batch of fuel. The study illustrates how a dissipating self‐assembled system can cope with the intermittent availability of chemical energy without compromising long‐term structural stability.

## Graphical abstract

Injection of a chemical fuel at a precise location causes a transition from thermodynamically stable to dissipative assemblies. After local depletion of fuel the system returns to the resting state. The study illustrates how a self‐assembled system can cope with the intermittent availability of chemical energy without compromising long‐term structural stability.

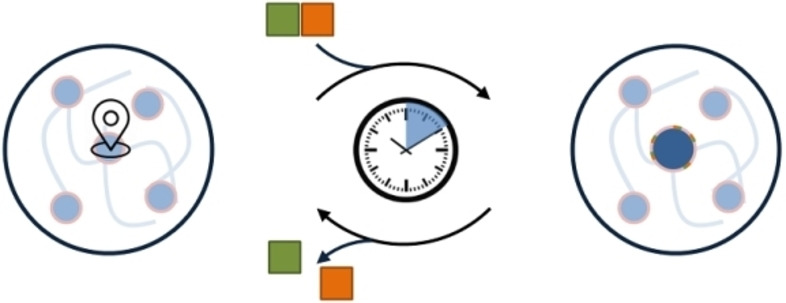

The transition from inactive to active matter is one of the defining moments in the emergence of life.[^1^, ^2^] At some moment in time prebiotic molecules came together to form organized structures.[^3^, ^4^, ^5^, ^6^] Self‐assembly, i.e. the thermodynamically controlled formation of supramolecular structures, is a powerful tool to assemble molecules, but the resulting structures reside at thermodynamic equilibrium and are consequently inactive (Figure 1a).^[7]^ The transition to active matter requires self‐organized non‐equilibrium structures able to extract energy from the environment to carry out work (Figure 1b).[^8^, ^9^, ^10^] The molecular understanding of the formation and functioning of non‐equilibrium structures has increased significantly.^[11]^ Initiating with the development of synthetic molecular machines,[^12^, ^13^] insights have emerged that the same physical‐organic principles that drive the non‐equilibrium operation of molecular devices are also at the basis of self‐organized structures both at the molecular and macroscopic level.[^14^, ^15^, ^16^, ^17^, ^18^] Chemically fueled self‐organized structures can emerge when an endergonic self‐assembly process is coupled to an exergonic fuel‐to‐waste reaction allowing a flow of energy between the two processes.[^14^, ^19^, ^20^, ^21^] Such a coupling can be established when a chemical fuel, a thermodynamically activated, but kinetically stable molecule,[^22^, ^23^] templates the formation of an assembly that is a catalyst for the fuel‐to‐waste reaction.[^24^, ^25^, ^26^, ^27^] Indeed, active matter in nature, with microtubules as the archetypical example,[^28^, ^29^] relies on the self‐organization of enzymes.^[16]^ The substrate‐templated assembly of catalytic structures has recently emerged as an attractive process for the development of active matter,^[30]^ because it inherently connects catalysis and the formation of dissipative structures.[^31^, ^32^, ^33^]

**Figure 1:**
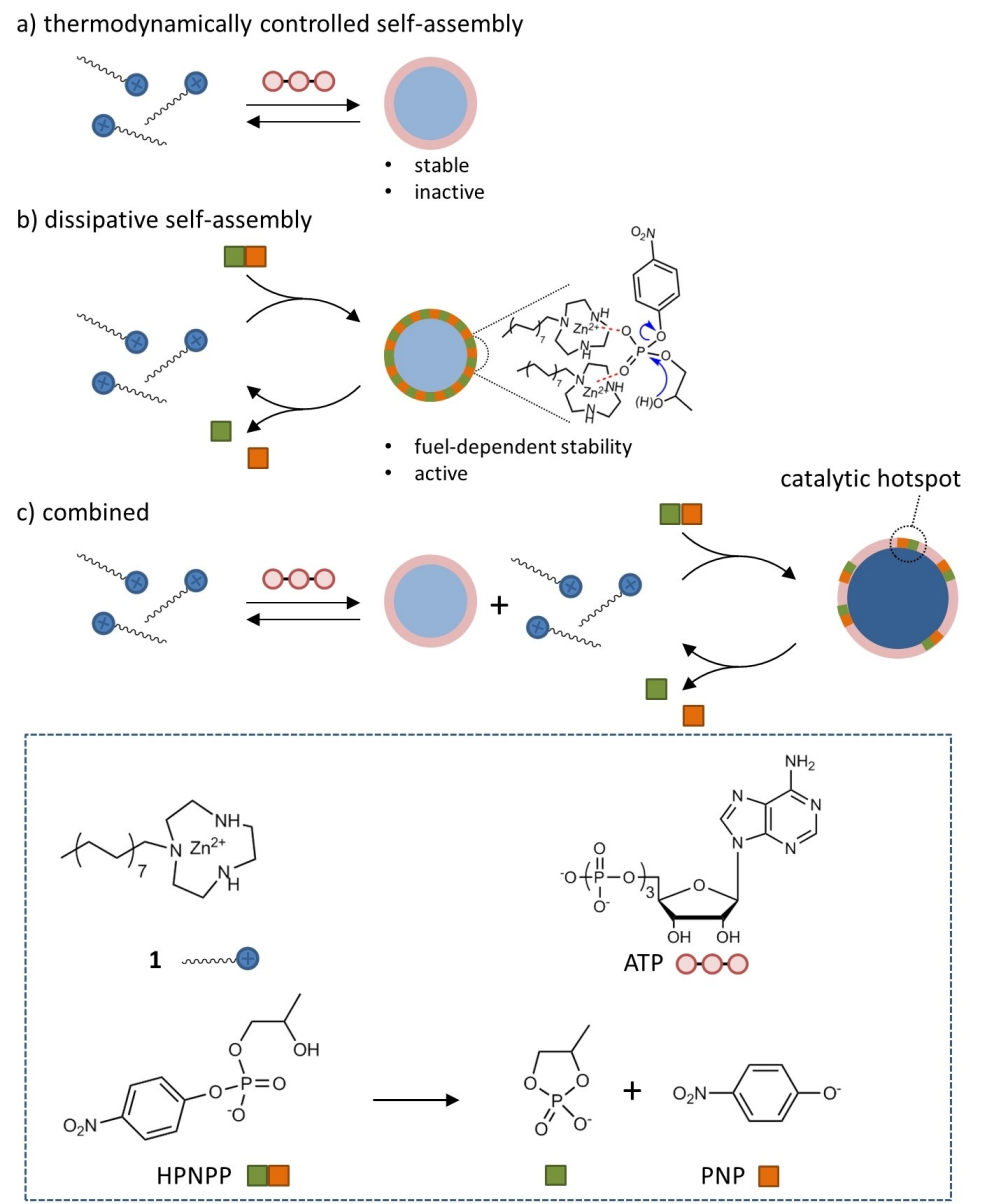
Schematic representation of thermodynamic and dissipative self‐assembly processes and a combination of both.

Self‐assembly provides structures that are thermodynamically stable but without the capacity to carry out work; the opposite is true for self‐organized structures. Dissipative structures can extract energy from the environment but the dependence of structural integrity on the continuous supply of energy makes the evolutionary emergence of dissipative structures a delicate process. It is therefore tempting to hypothesize that systems relying on a combination of self‐assembly and self‐organization processes may have played an important role in the transition from inactive to active matter (Figure 1c). Over the past years a large number of chemically‐fueled assembly processes have been described,[^24^, ^25^] but in nearly all cases the exclusive presence of an energy dissipating process makes the system shuttle between a disassembled and assembled state.

Recently,^[34]^ we have described a different system that combines both thermodynamic and dissipative processes: the presence of adenosine‐triphosphate (ATP) templated the self‐assembly of surfactant molecules **1**, containing 1,4,7‐triazacyclononane (TACN)⋅Zn(II)‐head groups, at concentrations well below the critical aggregation concentration of **1** alone. The presence of thermodynamically stable ATP‐templated assemblies facilitated the activation of a dissipative self‐assembly process in which the substrate 2‐hydroxypropyl *p*‐nitrophenylphosphate (HPNPP) caused the inclusion of additional surfactant **1** in the ATP‐templated assemblies. The facilitation of the HPNPP‐templated assembly is attributed to the lower entropy cost for the HPNPP‐templated insertion of **1** into preorganized ATP‐templated assemblies as compared to the entropy cost associated with the formation of assemblies of **1** templated just by HPNPP. Enhanced binding of a weak binder in the presence of a strong binder has also been observed in other systems.[^35^, ^36^]

Interestingly, these additional surfactants self‐organized into ‘catalytic hotspots’ that catalysed the conversion of HPNPP as a result of the cooperative action between neighbouring Zn(II)‐complexes. The reliance on both thermodynamic and dissipative processes renders the system an interesting model for studying the transition from inactive to active matter particularly related to the question of structural integrity in response to the intermittent availability of energy. We could not address this question in our previous studies in solution because the high concentrations of HPNPP substrate required to induce the formation of hotspots did not permit repetitive additions of substrate because of waste interference. Here, we demonstrate that this problem can be solved by using agarose hydrogel as a reaction medium. The hydrogel matrix blocks mass transport through convection enabling an exploitation of the difference in diffusion rates between small and large structures to install and maintain concentration gradients that persist in time.[^37^, ^38^, ^39^, ^40^] We have recently shown that the local injection of surfactant **1** in an agarose hydrogel containing HPNPP results in the local formation of dissipative assemblies that persist in time with continuous diffusion of HPNPP to the assemblies and waste molecules diffusing away.^[41]^ Here, we show that by operating the ATP‐ and HPNPP templated processes together in a hydrogel matrix 1) catalytic hotspots spontaneously form upon the local activation of an ATP‐templated self‐assembly process, and 2) structures can indeed switch transiently from a thermodynamic to a dissipative state when a batch of chemical fuel is administered.

Our initial studies were aimed at demonstrating that in a hydrogel catalytic hotspots can form in a spontaneous manner as a result of diffusion of surfactant **1** (Figure 2a). A reference agarose gel (1 mg/ml, buffered at pH 7.0) containing a homogeneous solution of **1** (100 μM) and HPNPP (125 μM) was prepared in 6‐well microtiter plates (*d*_well_=3.5 cm) (Figure 2b). At these concentrations HPNPP templates the formation of assemblies **1**⋅HPNPP as evidenced by the presence of small assemblies (*d*∼23±6 nm) in TEM images (Figure 2e, Figure S12a) of samples taken from the gel.^[31]^ The dissipative nature of these assemblies is evidenced by the gradual increase in absorbance at λ=405 nm over time as a result of an increase in *p*‐nitrophenolate concentration (Figure 2c). Although just the increase for positions 1 and 5 is shown, it is noted that the absorbance increased homogeneously all over the gel. No increase in absorbance was observed when the gel contained just HPNPP (Figure S3).

**Figure 2:**
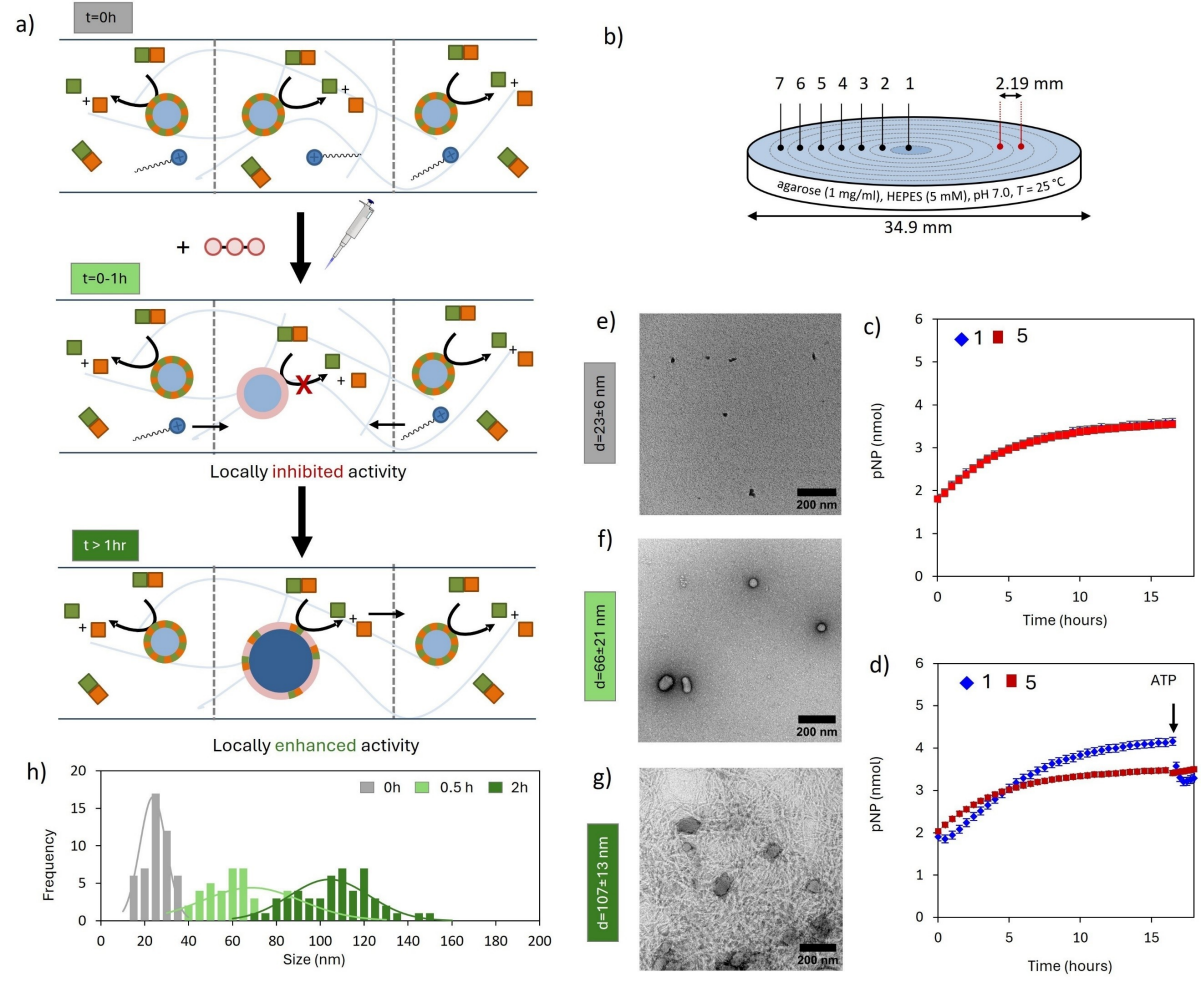
(a) Schematic representation of the chemical processes that occur in a gel containing **1** and HPNPP upon the injection of ATP. (b) Experimental set up for local activation. The numbers indicated in the gel refer to the areas of which the absorbance intensity values are measured. Experimental data for areas 1 and 5 are reported in Figures 2c and d. A full account is given in Figure S4. (c) Absorbance at 405 nm for positions 1 and 5 as a function of time for a gel containing **1** (100 μM) and HPNPP (125 μM). (d) Absorbance at 405 nm for positions 1 and 5 as a function of time after the injection of 1 μL of a 5 mM stock solution of ATP in the position 1 of a gel containing **1** (100 μM) and HPNPP (125 μM). After 17 h, 1 μL of a 5 mM stock solution of ATP was injected in position 1. Transmission electron microscopy (TEM) images of position 1 of the gel in which ATP was injected taken at time=0 h (e), 0.5 h (f), 2 h (g). Additional TEM images are provided in section 8 of the Supporting Information. The scale bars correspond to 200 nm. (h) Size distribution of structures observed in TEM images (see also section 8 and 11 of the Supporting Information), fitted with normal distribution function (represented with solid line). Experimental conditions: agarose=1 mg/ml, [**1**]= 100 μM, [HPNPP]= 125 μM, [HEPES]=5 mM, *T*=25 °C. Error bars indicate the standard deviation calculated from duplo measurements.

To study the formation of catalytic hotspots, a gel with the same composition ([**1**]=100 μM and [HPNPP]=125 μM) was prepared, but this time subjected to the injection of a tiny volume (1 μL) of a highly concentrated solution of ATP (5 mM) in the center of the gel (position 1, Figure 2b). Comparison of the absorbance increase at different distances from the center revealed strong differences. Whereas the absorbance increase at large distances (*e.g*. position 5) from the injection point followed the same kinetics as the one observed for the reference gel (Figure 2d—red trace), the increase in the center followed a different profile (Figure 2d—blue trace). No increase was observed in the first hour, but then the absorbance started to increase with a rate that was higher than the other positions and the reference gel. Even after 17 hours a significantly higher catalytic activity was still observed in the center where ATP had been injected compared to surrounding positions. The approximately constant absorbance in position 1 after 17 hours indicates that at that time the amount of PNP produced through local catalysis equals the amount of PNP diffusing away.^[41]^ Indeed, catalytic activity after 17 hours was confirmed by the observation that the injection of a new batch of ATP, a strong inhibitor, resulted in a decrease in absorbance in position 1 (Figure 2d). The decrease in absorbance is explained by the inhibition of the catalytic production of PNP by the newly added ATP, whilst diffusion of the excess of PNP from position 1 to the surroundings continues. Indeed, the injection of ATP at the catalytically inactive position 5 did not cause any change in local absorbance (Figure S6).

TEM analysis revealed that the injection of ATP resulted in structural changes in the assemblies. A sample taken from the center at t=30 min, that is when catalysis was inhibited, revealed spherical assemblies with a size of around 66±21 nm (Figure 2f, Figure S12b), which is significantly larger than the size of assemblies **1**⋅HPNPP observed before injection (*d*∼23±6 nm, Figure 2e, Figure S12a). However, a sample taken from the center at t=2 h, that is after the absorbance had started to increase, revealed that assemblies had become much larger with a less regular shape (*d*=107±13 nm, Figure 2g, Figure S12c).

These observations can be explained with our knowledge of this system based on previous solution studies (Figure 2a).^[34]^ Injection of ATP, a template with a much higher affinity for **1** compared to HPNPP, results in the local formation of ATP‐templated assemblies **1**⋅ATP, which locally depletes both **1**⋅HPNPP and unassembled **1** and consequently causes inhibition of catalysis. The size of the assemblies 0.5 h after injection corresponds indeed to the size expected for assemblies **1**⋅ATP.^[42]^ Since the total amount of injected ATP is very small, only a small central portion of the gel is affected. We have previously shown that assemblies **1**⋅ATP remain localized at the injection point because their large size imparts a low diffusion coefficient.^[37]^ On the other hand, the depletion of unassembled **1** in the center causes a macroscopic concentration gradient for unassembled **1** which subsequently diffuses from the surroundings towards the center. Evidence for the diffusion of **1** to the center was obtained from ICP‐MS, which showed an increase in the concentration of Zn^2+^ ions from 103 to 138 μM 10 hours after injection of ATP (Figure S7). No variation in Zn^2+^‐concentration was observed for remote position 5 of the same gel or position 1 of a gel into which ATP was not injected. Following diffusion of **1** to the center, the coexistence of assemblies **1**⋅ATP, unassembled **1** and HPNPP in the center creates the conditions for the formation of catalytic hotspots, i.e. HPNPP templates the insertion of additional **1** in assemblies **1**⋅ATP which reactivates catalysis. The activation of a substrate‐induced dissipative self‐assembly process is supported by the presence of much larger assemblies after the onset of catalysis. The remarkable observation is that the new rate is higher than the one observed before injection of the ATP (Figure 2c and 2d). This implies that catalysis resulting from the HPNPP‐induced templation of **1** benefits from the presence of assemblies **1**⋅ATP. Further support for this interpretation came from control experiments in which lower amounts of ATP were injected. Reduced quantities of ATP resulted both in a reduction of the lag time and a reduced rate enhancement (Figure S4 and Figure S5). We attribute the increase in lag time for higher concentrations of injected ATP to the higher amount of surfactant that has to diffuse to the center to create the optimal ratio of **1**:ATP for the formation of catalytic hotspots. An alternative explanation that the increase in catalytic activity is simply a result of the accumulation of **1** in the center of the gel is discarded, because it does not consider that a large part of **1** is not available for catalysis because of the interaction with ATP, which acts as a strong inhibitor (Figure S9).

We then focused our attention on the key question of this study, which regards the extent to which the presence of thermodynamically stable ATP‐templated assemblies makes the structural integrity of the system resilient towards an intermittent availability of chemical fuel (Figure 3a). To address this question agarose gels (1 mg/ml, buffered at pH 7.0) were prepared containing surfactant **1** (100 μM) and three different concentrations of ATP (0, 5 and 20 μM); the idea being that the presence of ATP would lead to the presence of homogeneously distributed stable ATP‐templated assemblies in the gel. To each gel a small volume of a concentrated HPNPP stock solution (1 μL, 90 mM) was injected and the increase in absorbance at 405 nm was monitored in time with spatial resolution (Figure 3b–d). A plot of the total HPNPP conversion in the gels is provided in Figure S10. In the reference gel without ATP an immediate increase in absorbance was observed in the center and subsequently also in the other positions of the gel with lag times that depended on the distance from the center (Figure 3b). The absorbance in the center reached a maximum after around 4 hours after which it gradually decreased. A plot of the conversion of HPNPP in the gel as a function of time (Figure S10) revealed no further increase after around 7 hours indicating that catalysis had stopped (30 % conversion) and that all further changes in absorbance were just a result of diffusion of PNP in the gel. After 20 hours the same absorbance was detected all over the gel indicating that unreacted substrate and waste had reached a homogeneous distribution. TEM analysis revealed that initially the system resided in a mostly unassembled state of **1**. Just a small number of tiny clusters of **1** (*d*=14±6 nm, Figure 3e) were observed. Two hours after the injection of HPNPP an increased number of assemblies of larger size (*d*=42±9 nm, Figure 3f) was observed, which had disappeared again after 15 hours (Figure 3g). Thus, by locally spiking the gel with HPNPP, assembly formation can be induced, but in time the system returns to the mostly unassembled resting state (Figure 3o).

**Figure 3:**
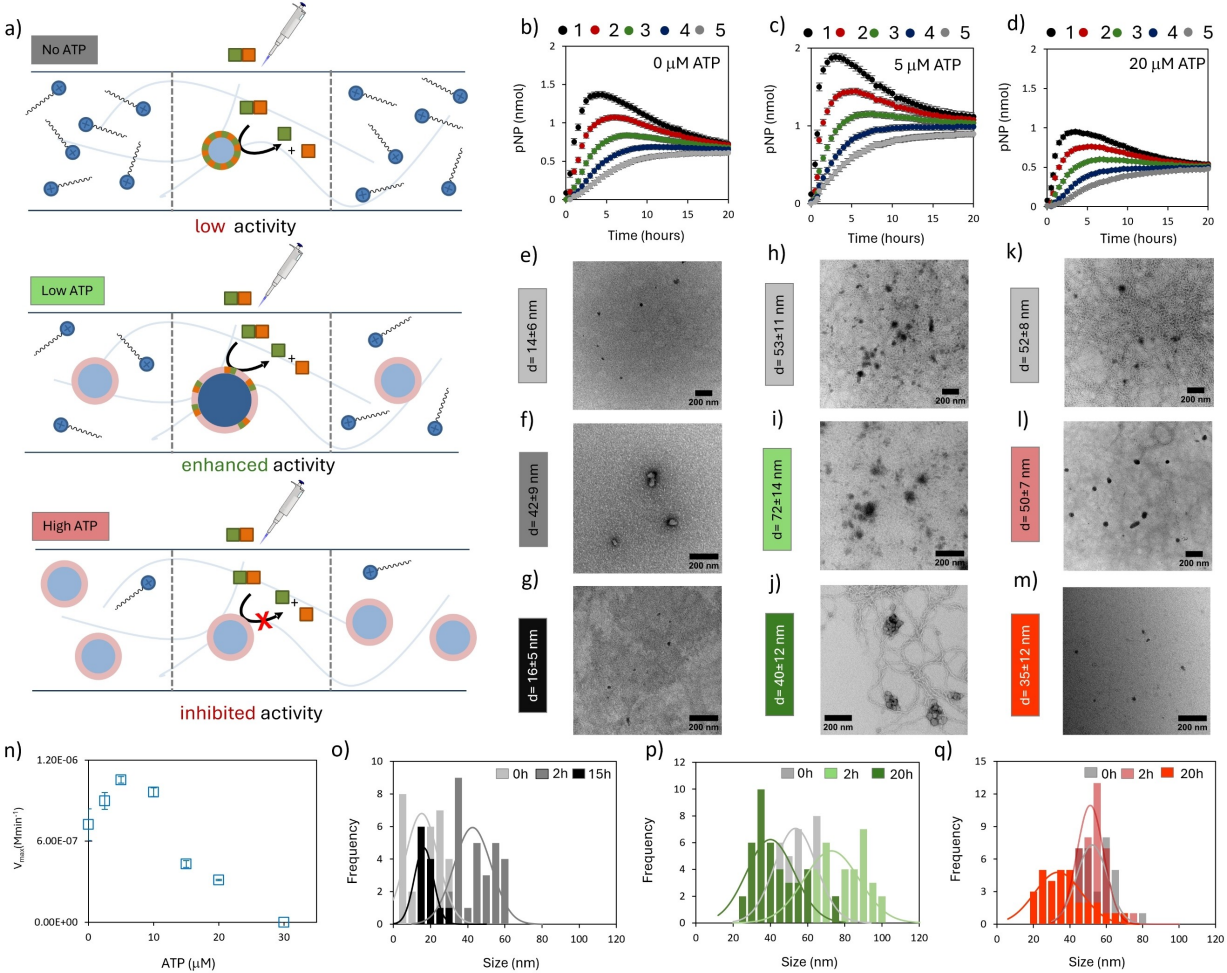
(a) Schematic representation of the processes that occur in a gel containing **1** in the absence of ATP (top), low concentration of ATP (5 μM, middle) and a high concentration of ATP (20 μM, top) upon the injection of same amount of HPNPP. In the top figure surfactant **1** is represented in an unassembled form in the absence of HPNPP. Considering the critical aggregation concentration of around 100 μM for **1** in solution^[42]^ and some stabilizing effect of agarose on assemblies of **1**,^[38]^ we anticipate **1** to be in a partially assembled state. Previous studies^[41]^ have shown that this does not block the diffusion of **1** through the matrix. b) Absorbance at 405 nm for positions 1–5 as a function of time after the injection of 1 μL of a 90 mM stock solution of HPNPP in the center of a gel containing **1** (100 μM). Transmission electron microscopy (TEM) images of position 1 of the gel taken at time=0 h (e), 2 h (f), 15 h (g). (o) Size distribution of structures observed in TEM images taken at different times, fitted with normal distribution function (solid line). Additional TEM images are provided in reference 41 (SI). c) Absorbance at 405 nm for positions 1–5 as a function of time after the injection of 1 μL of a 90 mM stock solution of HPNPP in the center of a gel containing **1** (100 μM) and ATP (5 μM). Transmission electron microscopy (TEM) images of position 1 of the gel taken at time=0 h (h), 2 h (i), 20 h (j). (p) Size distribution of structures observed in TEM images taken at different times, fitted with normal distribution function (solid line). Additional TEM images are provided in section 9a of the Supporting Information. d) Absorbance at 405 nm for positions 1–5 as a function of time after the injection of 1 μL of a 90 mM stock solution of HPNPP in the center of a gel containing 1 (100 μM)+ATP (20 μM). Transmission electron microscopy (TEM) images of position 1 of the gel taken at time=0 h (k), 2 h (l), 20 h (m). (q) Size distribution of structures observed in TEM images taken at different times, fitted with normal distribution function (solid line). Additional TEM images are provided in section 9c of the Supporting Information. The scale bars in all TEM images correspond to 200 nm. n) Maximum rates of HPNPP hydrolysis (t=10–50 minutes) as a function of the initial amount of ATP present in the gel with [**1**]=100 μM. Experimental conditions: agarose=1 mg/ml, [HEPES buffer]=5 mM, *T*=25 °C. Error bars indicate the standard deviation calculated from duplo measurements.

Major differences were observed when low concentrations of ATP (5 μM) (Figure 3c) were present in the gel. Compared to the reference gel, injection of the same amount of HPNPP in the gel led to a faster increase in absorbance in position 1 and a higher maximum absorbance before the system gradually reached equilibrium after 20 hours. At this point the cycle could be repeated by injecting a second batch of HPNPP (Figure S11). This important observation shows the advantage of using a hydrogel because, as mentioned earlier, refueling could not be achieved in solution.^[34]^ The higher initial rate and the increased overall conversion of HPNPP after 20 hours (45 %) indicated that the gel is catalytically more active when 5 μM of ATP is present (Figure S8 + Figure S10). Also differences in the structural evolution were detected. The presence of ATP templates the formation of assemblies **1**⋅ATP (*d*=52±8 nm, Figure 3h, Figure S13a) which are detected in large number before injection of HPNPP. Two hours after injection of HPNPP the assemblies in position 1 had significantly increased in size (*d*=72±14 nm, Figure 3i, Figure S13b). However, the increase in size was temporary, because after 20 hours the assembly size had returned to the original distribution (Figure 3j, Figure S13C). The transient increase in assembly size[^43^, ^44^, ^45^] is attributed to the transient presence of a high HPNPP concentration in position 1 which induces the HPNPP‐templated insertion of additional **1** in assemblies **1**⋅ATP (Figure 3p). Indeed, sampling the assembly size at a remote position from the center revealed no changes is size over the same time interval (Figure S14). Furthermore, assemblies in gels in which the same amount of HPNPP or a 50/50 mixture of HPNPP and waste was distributed homogeneously all over the gel showed assemblies with a size corresponding to assemblies **1**⋅ATP (Figure S16). In summary, the transient availability of substrate causes a temporal transition from thermodynamically stable to dissipative assemblies. After local depletion of the substrate by catalysis and diffusion the structures return to the thermodynamically stable resting state.The third gel with a higher concentration of ATP (20 μM) revealed a lower catalytic activity in position 1 compared to the reference gel without ATP and also a lower overall conversion after 20 hours (Figure 3d and Figure S10). Furthermore, no transient increase in assembly size could be detected; at the three checkpoints (t=0, 2 and 20 h) the assembly size distribution remained centered at around 50 nm (Figure 3k–m, Figure 3q, Figure S15). These observations indicate that the ability of the system to respond to HPNPP by transiently increasing the assembly size with concomitant increase in catalytic activity strongly depends on the ratio of **1**:ATP present in the gel. To get a full account, we measured the catalytic activity upon the injection of HPNPP in gels with additional ATP concentrations covering the ATP‐concentration range between 0 and 30 μM. A plot of the initial rate in position 1 as a function of the ATP concentration revealed a maximum for 5 μM ATP, whereas at 30 μM ATP catalysis was completely inhibited (Figure 3n, Figure S10). The bell‐shaped curve is in agreement with the solution data reported before^[34]^ and confirms the surprising observation that the presence of small amounts of ATP, which, as reminded, has a much stronger affinity for **1** compared to HPPNP, facilitates catalysis rather than inhibiting it. This effect can only be observed under conditions where the available amount of ATP is low enough to ensure the contemporary presence of unassembled **1** alongside assemblies **1**⋅ATP.

In conclusion, we have shown how the presence of a thermodynamically controlled self‐assembly process facilitates the activation of a dissipative self‐assembly process in which a substrate templates the self‐assembly of a catalytic pocket for its own destruction. Spontaneous activation of the dissipative self‐assembly process is observed in two different scenarios. In case ATP is locally administered to a gel containing surfactant and substrate, activation is a result of spontaneous diffusion of unassembled surfactant to the place where ATP was injected. The important observation in this scenario is that overall the system becomes catalytically more efficient in the presence of a molecule (ATP) that provides a thermodynamically stable structure. Catalytic activity increases even though catalytic units are subtracted by ATP, which is a relevant phenomenon to be considered in the design of multivalent self‐assembled receptors and catalysts.^[46]^ In the second scenario, catalytic hotspots are locally activated, but for a limited duration, by administering substrate to the gel. It is tempting to contextualize this scenario in the context of the evolutionary transition from inactive to active matter. Thermodynamically controlled self‐assembly ensures long‐time structural stability, but also creates favorable conditions for the activation of an energy‐dependent dissipative self‐assembly process. The disconnection between structural stability and energy consumption allows the structure to ‘survive’ periods during which energy is not available. Additionally, the results show that the energy‐dissipating process is more efficient when thermodynamically stable assemblies are present in the system.

## Supporting Information

Procedures, experimental details and supporting data is available. The authors have cited an additional reference within the Supporting Information.^[47]^

## Conflict of Interests

The authors declare no conflict of interest.

## Supporting information

As a service to our authors and readers, this journal provides supporting information supplied by the authors. Such materials are peer reviewed and may be re‐organized for online delivery, but are not copy‐edited or typeset. Technical support issues arising from supporting information (other than missing files) should be addressed to the authors.

Supporting Information

## Data Availability

The data that support the findings of this study are available from the corresponding author upon reasonable request.
