# Supplementary material for: Transient transition from Stable to Dissipative Assemblies in Response to the Spatiotemporal Availability of a Chemical Fuel
Source: Angew Chem Int Ed Engl. 2024 Nov 11;64(2):e202414495. doi: 10.1002/anie.202414495 (PMC11720371; doi:10.1002/anie.202414495)
Supplement: Supplementary file 1 — Supporting Information [file ANIE-64-e202414495-s001.pdf]

Angewandte Chemie
Eine Zeitschrift der Gesellschaft Deutscher Chemiker

Supporting Information

Transient transition from Stable to Dissipative Assemblies in Response to the Spatiotemporal Availability of a Chemical Fuel

*H. Kar, R. Chen, K. Das, L. J. Prins**

Supporting Information

Transient transition from stable to dissipative assemblies in response to the spatiotemporal availability of a chemical fuel

Haridas Kar, Rui Chen, Krishnendu Das, Leonard Jan Prins*

Department of Chemical Sciences, University of Padua, Via Marzolo 1, 35131 Padua, Italy

Table of Contents

Entry	Details	Page No
1.	Materials and instrumentation	SI - 2
2.	Gel preparation	SI - 3
3.	Data acquisition and analysis	SI - 4
4	ATP injection experiments	SI – 6
5.	ICP-MS measurements	SI – 7
6.	HPNPP injection experiments	SI – 9
7	Repetitive injections of HPNPP	SI – 12
8.	Characterization of self-assembled structures by TEM (ATP injection)	SI – 13
9.	Characterization of self-assembled structures by TEM (substrate injection)	SI – 14
10.	TEM images of a gel containing homogenously distributed substrate and waste	SI – 17
11.	Tables containing the size of assemblies and distributions	SI – 18
12.	References	SI – 24

1. Materials and instrumentation

Materials

Low electroendosmosis (EEO) agarose, buffer 4-(2-hydroxyethyl)-1-piperazineethanesulfonic acid (HEPES), and reagents for synthesis were purchased from Merck. UV-Vis and fluorescence measurements were carried out using deionized water filtered by a Milli-Q water purifier (Millipore). The 6-well glass bottom plates were purchased from Cellvis and Greiner multiwell plate sealers were purchased from Merck.

Adenosine 5'-triphosphate (ATP) disodium hydrate sodium salt stock solutions were prepared in MilliQ water by weight and the exact concentration was calculated by UV-Vis spectroscopy using the molar extinction coefficient: ε_{259} (ATP) = 15400 M^{-1} cm^{-1}.

A measured amount of $Zn(NO_3)_2$ was dissolved in MilliQ water to obtain a stock solution and a concentration of Zn of 435 mM was determined by ICP (inductively coupled plasma) analysis.

The synthesis and characterization of C_{16}TACN (**1**) and HPNPP have been reported. [1, 2]

Instrumentation

pH Measurements: The pH of buffer solutions was determined at room temperature using a Metrohm-632 pH meter equipped with an Ag/AgCl/KCl reference electrode and calibrated with standard buffer solutions at pH 7.00.

Plate reader: Time-dependent absorbance and fluorescence readings were measured on a TECAN (M1000) PRO plate reader.

TEM: Dry-state-stained transmission electron microscopy (TEM) was performed on a FEI Tecnai G2 electron microscope with an acceleration voltage of 120 kV. The instrument was equipped with a OSIS Veleta 4k CCD camera. The TEM-grid surface was cleaned and made hydrophilic with glow discharge by Leica EM ACE600. First, the grid was placed on a drop of sample gel (~10 µL), for 1 min. Excess sample was blotted from each grid using filter paper and the grid was subsequently stained by depositing an 10 µL droplet of uranyl acetate (2 %) for 30 seconds. The stained sample was blotted again, before drying at room temperature. Images were analyzed using Image J software.

2. Gel preparation

For the preparation of HEPES-buffered agarose gels (3 mL) containing HPNPP, 3 mg of agarose was weighted in a 4 ml vial and dissolved in Milli-Q water using mild heating (closed vial). After all agarose had dissolved, the transparent homogeneous solution was left at room temperature to cool until 40°C (approximately 3 minutes) after which HEPES-stock solution (150 μL) was added.

ATP injection: 75 μL of a $C_{16}TACN\cdot Zn^{2+}$ stock solution (4 mM, final concentration 100 μM) and 18.8 μL of a HPNPP stock solution (20 mM, final concentration 125 μM) were added to the buffered agarose solution (1 mg/ml). After gentle shaking, the solution was quickly transferred to a 6-well glass bottom microtiter plate. After solidification of the gel (~2h), 1 μL of ATP stock solution with different concentrations (0.5 mM to 5 mM) was injected in the centre of the gel. A sealing film was applied to cover the well to avoid evaporation during the measurements. The changes in the absorbance at 405 nm were followed over time.

Substrate Injection: 75 μL of a $C_{16}TACN\cdot Zn^{2+}$ stock solution (4 mM, final concentration 100 μM) was added to the buffered agarose solution (1mg/ml). Different amounts of ATP (concentration ranges from 2.5 μM to 30.0 μM) were then added to the solution After gentle shaking, the solution was quickly transferred to a 6-well glass bottom plate after. A transparent gel was obtained after 2 h of solidification at room temperature. After that, 1 μL of a HPNPP stock solution (90 mM) was injected in the centre of gel. A sealing film was applied to avoid evaporation. The changes in the absorbance at 405 nm were followed over time.

Supplementary Table 1. Volumes used to prepare agarose gels with different ATP concentrations

Entry	Amount of ATP (stock solution= 1 mM) (μL)	HEPES (stock solution 100 mM, final concentration 5 mM) (μL)	Milli Q water (μL)
0 μM ATP	0	150	2775
2.5 μM ATP	7.5	150	2767
5.0 μM ATP	15.0	150	2760
10.0 μM ATP	30	150	2745
15.0 μM ATP	45	150	2730
20.0 μM ATP	60	150	2715
30.0 μM ATP	90	150	2685

3. Data acquisition and analysis

Data collection and analysis: Measurements were performed at 177 points of each well, distributed as shown in Figure S1. The external area of the well (1 mm from the border) was excluded to avoid border effects. The data points were grouped in 7 areas, corresponding to 7 concentric circles around the injection point (see color scheme in Figure S1). The distance between two adjacent circles is 2.19 mm (see Figure 2b in the manuscript). In the manuscript we have reported the changes in absorbance and fluorescence data corresponding to positions 1-5.

The different thickness across the gel in microplates affects the optical pathway and consequently the measured signal intensity. Following the abovementioned grouping of the data points into the eight sections, a normalization factor was determined for each location with respect to the starting value of position 7 (i.e., referring to a gel in which components are homogeneously distributed and concentration gradients are absent).[S3] The obtained normalization factors were then used to correct for each position the measured absorbance intensities obtained during the kinetics.

Each gel experiment was repeated twice to minimize errors. The measured absorbance values were normalized on the value at time 0, and average values were considered.

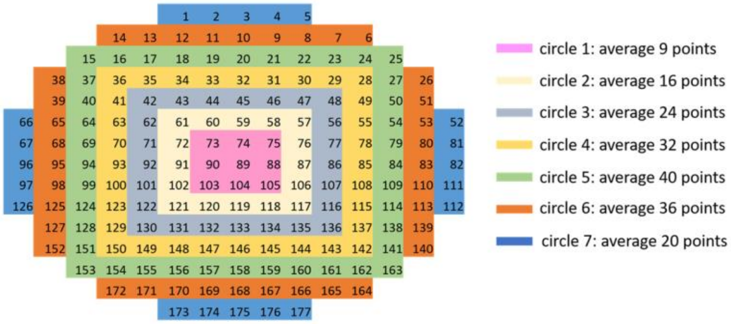

Figure S1. Example of collected data for a single well of a 6-well glass bottom microtiter plate. Multiple positions (177 points) were read for each well. The obtained value for each circle (referred to in the manuscript as position) was an average of multiple reads.

Substrate conversion: To determine substrate conversion (HPNPP) in the gel, a calibration curve of *p*-nitrophenol (moles vs total absorbance) was constructed. The absorbance values measured for different concentrations of *p*-nitrophenol were subtracted from the background (i.e., buffered agarose gel). By summing the absorbance in positions 1-7 the total absorbance was calculated. The total absorbance vs moles (different concentrations) curve was constructed and fitted in excel (Fig. S2b). All absorbance data obtained during the kinetic runs was corrected for background and for each data set the total absorbance value was converted into moles using the calibration curve. The substrate conversion was then calculated by dividing by the initial moles of substrate present and multiplying with 100.
The maximum rates for HPNPP hydrolysis were calculated from the absorbance vs PNP concentration curve.

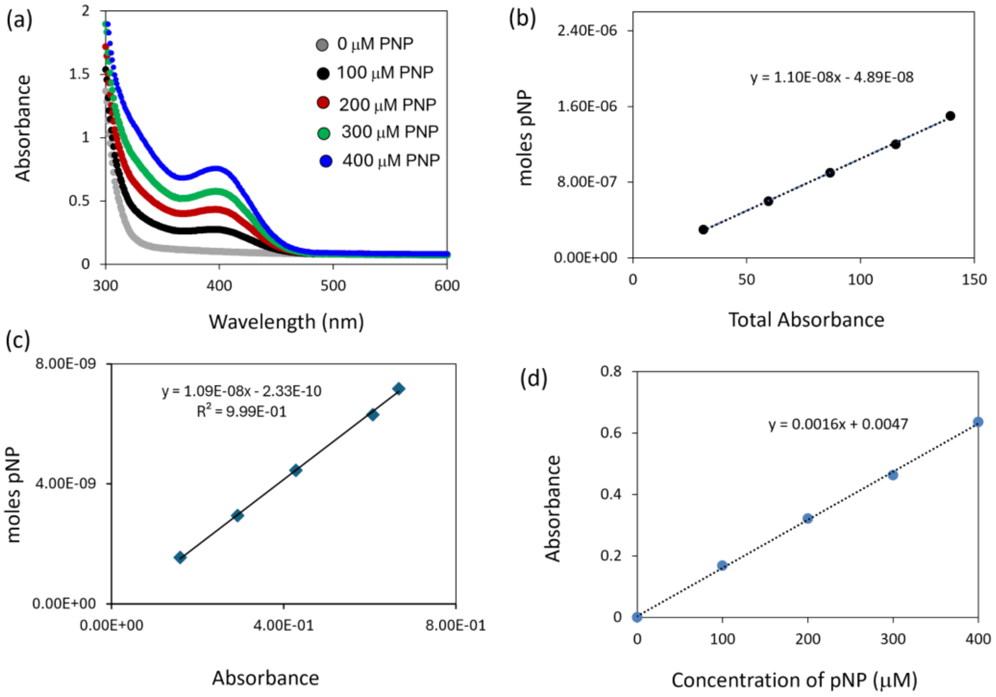

Figure S2. (a) UV/vis absorption spectra of *p*-nitrophenol at the central position in gel measured by plate reader. (b) Calibration curve plotting the moles of PNP in the gel as a function of the total absorbance at 405 nm obtained by summing the absorbance in positions 1-7 (after background subtraction). (c) Calibration curve plotting the moles of PNP vs absorbance at 405 nm in a single position. (d) The absorbance of *p*-nitrophenol in function of its concentration in gel recorded by plate reader (initial absorbance i.e; 0 μM PNP was subtracted). [HEPES] = 5 mM, pH 7, agarose= 1 mg/ml, T= 25°C.

Substrate hydrolysis in the absence of surfactant: Agarose gels (1 mg/ml) with a fixed amount of HPNPP (100 μM) were prepared and the absorbance band at 405 nm for positions 1-5 was measured as a function of time.

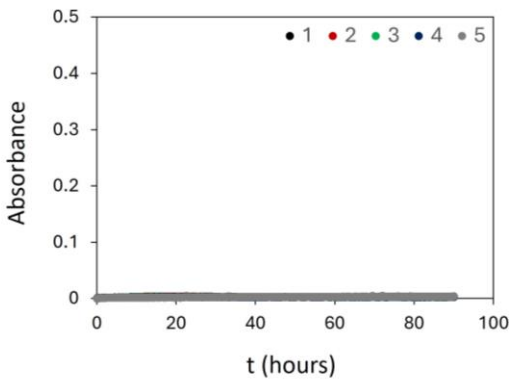

Figure S3. (a) Absorbance at 405 nm for positions 1-5 as a function of time was measured by microplate reader. [HPNPP]= 100 μM; [HEPES] = 5 mM, pH 7, agarose= 1 mg/ml, T= 25°C

4. ATP injection experiments

4a. Injection of ATP in position 1

The formation of catalytic hotspots in agarose gels was studied by injecting a tiny volume of ATP. A series of gels containing a fixed amount of surfactant **1** (100 μM) and substrate HPNPP (125 μM) were prepared. 1 μL of an ATP stock solution (concentration ranges 0.5 mM to 5.0 mM) was injected in the center of the gel. The absorbance at 405 nm for positions 1-5 was monitored as a function of time. To understand the effects of ATP injection, a control experiment was carried out using a reference gel in which ATP was not injected. Comparison of the different gels showed a clear effect of the presence of ATP (Figure S4).

Comparison of the lag time (defined as the period between ATP injection and the increase in absorbance at position 1) shows longer lag times for increased amounts of ATP (Figure S5). In this experimental set up (gel containing **1** and substrate) we attribute the increase in lag time to the higher amount of surfactant that needs to diffuse to the center to create the optimal ratio for the formation of catalytic hotspots.

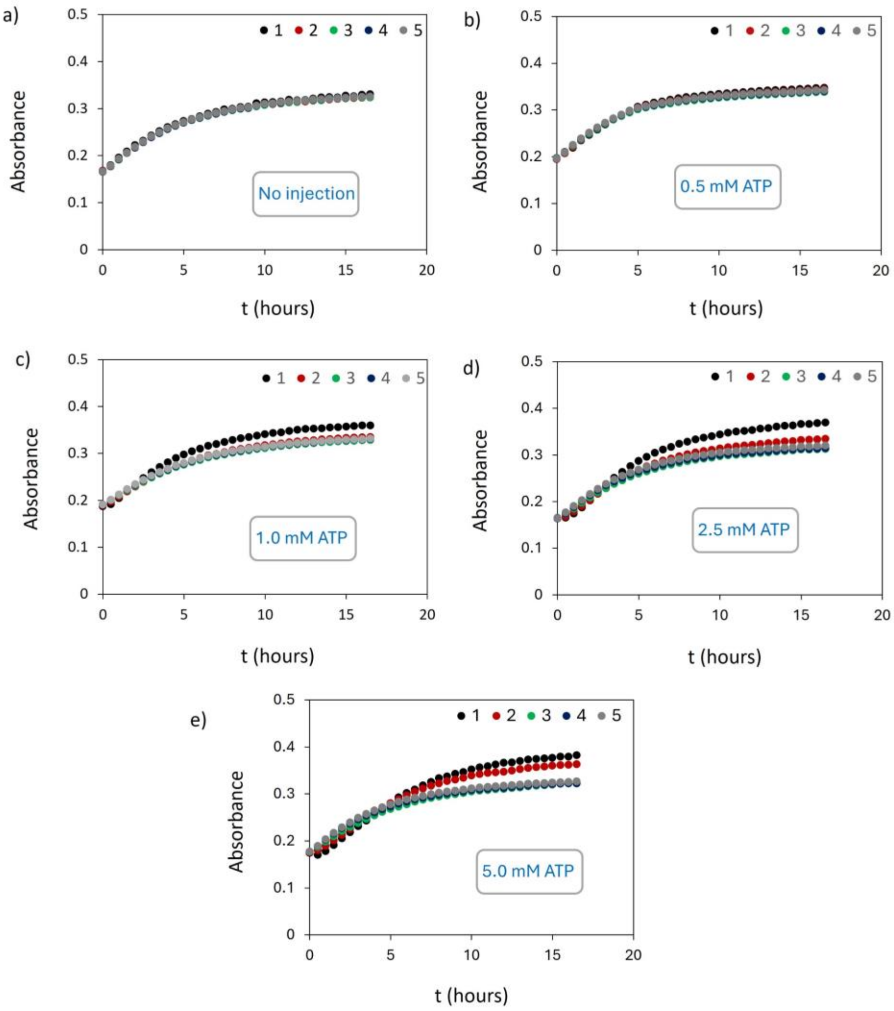

Figure S4. Absorbance at 405 nm for positions 1-5 as a function of time after the injection of 1 μL of stock solutions containing different amounts of ATP - [a) blank, b) 0.5 mM, c) 1.0 mM, d) 2.5 mM, e) 5.0 mM)] - in position 1 of gels containing surfactant **1** (100 μM), HPNPP (125 μM), [HEPES] = 5 mM, pH 7, agarose = 1 mg/ml, T= 25°C.

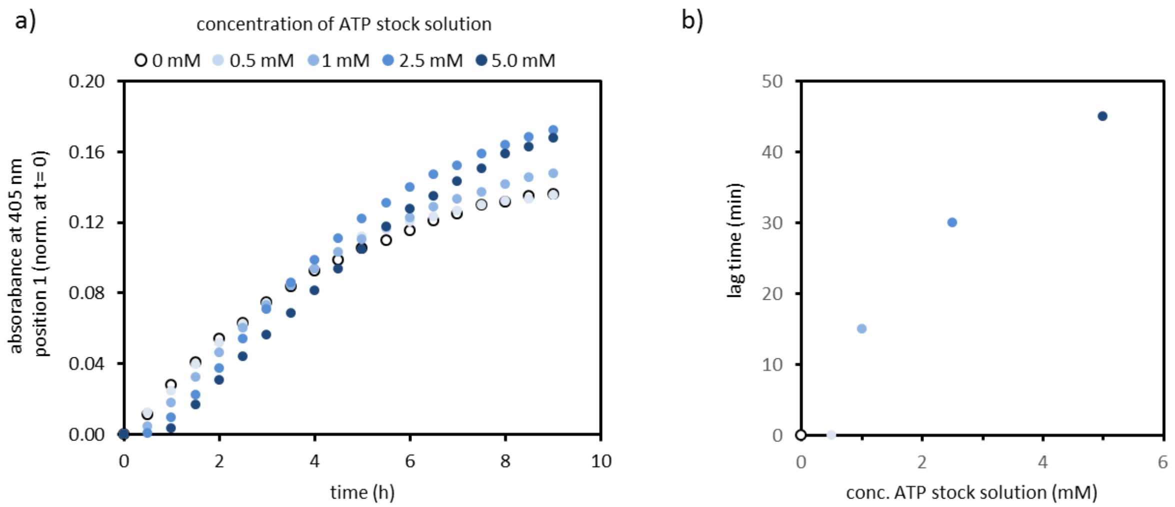

Figure S5. a) Absorbance (405 nm) in position 1 (norm. on t = 0) as a function of time upon the injection of 1 µl of ATP stock solutions at different concentrations. b) Lag time as a function of the concentration of the injected ATP stock solution. Experimental conditions: **1** (100 µM), HPNPP (125 µM), agarose= 1 mg/ml, [HEPES] = 5 mM, pH 7, T= 25°C.

4b. Confirmation of catalytic activity after 17 hours

To confirm ongoing catalytic activity due to the formation of catalytic hotspots, ATP (1 µL of a 5 mM stock solution) was injected in the center of the gel after 17 hours (Figure 2d of the manuscript). As a control experiment, ATP was also injected in the position 5 which is catalytically inactive after 15 hours (Fig. S5). In this case, just a very minor change in the absorbance was detected evidencing that the drop in absorbance in position 1 upon ATP injection results from the inhibition of catalysis.

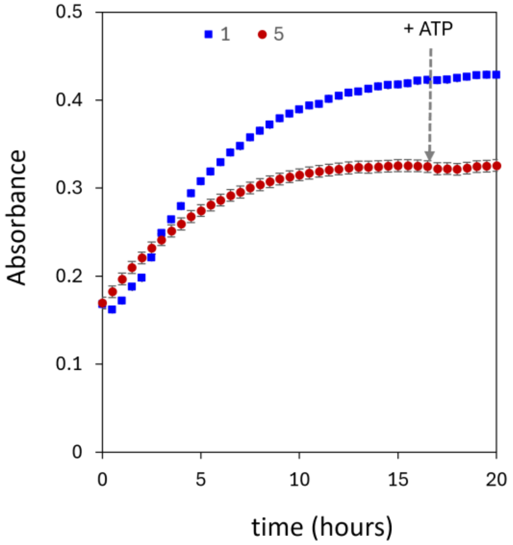

Figure S6. Absorbance at 405 nm for positions 1 and 5 as a function of time after the injection of 1 µL of a 5 mM stock solution of ATP in the center of a gel containing surfactant **1** (100 mM) and HPNPP (125 µM). After 14h, 1 µL of a 5 mM stock solution of ATP was injected in position 5. [HEPES] = 5 mM, pH 7, agarose= 1 mg/ml, T= 25°C.

5. Determination of surfactant 1 concentration after the injection of ATP

We determined the surfactant concentration by measuring the Zn^{2+} concentration using inductively coupled plasma mass spectrometry (ICP-MS). A series of agarose gels (1 mg ml^{-1}, buffered at pH 7.0) matrix were prepared with 100 μM **1** + 125 μM HPNPP. A measured amount of ATP (1 μL of a 5 mM stock solution) was injected at position 1 and samples were collected from position 1 and position 5 (see Figure 2b) at 0 h and 10 h. A control gel was prepared in which ATP was not injected. For the preparation of each sample a 100 μL aliquot was taken using a Gilson pipette and transferred directly into a 15 mL Falcon tube. Subsequently, 180 μL of HNO_3 (69%) was added, and the solution was digested in a bain-marie (100°C) for 1 hour. After digestion, the samples were cooled to room temperature, and milliQ water was added to reach 5 g. All samples were prepared in triplo.

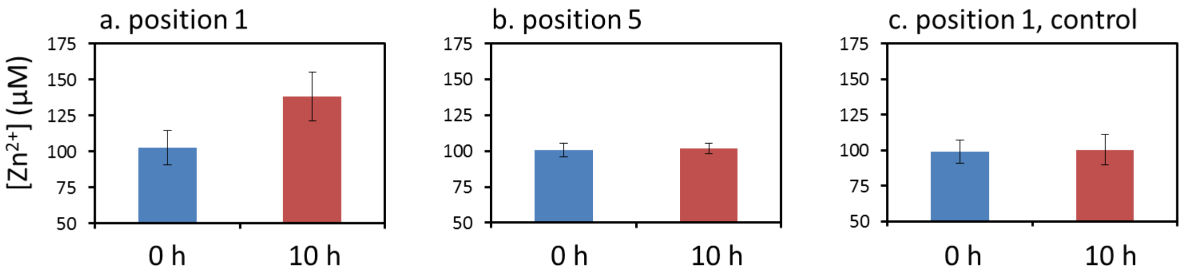

Figure S7. Concentration of Zn^{2+} (μM) for a) position 1, b) position 5 at t= 0h and 10h after the injection of 1 μL of a 5 mM stock solution of ATP in position 1. c) Concentration of Zn^{2+} at t= 0h and 10h of a control gel in which ATP was not injected. Experimental conditions: [**1**] = 100 μM, [HPNPP]= 125 μM, agarose = 1 mg/ml, [HEPES] = 5 mM, T = 25 °C.

Supplementary Table 2. Concentration of Zn^{2+} at t = 0 and 10h determined by ICP-MS

**Gels to which 1 μL of a 5 mM stock solution of ATP was injected**						
Sample	Time (h)	Zn^{2+} (μM)			Average	Stdev
Position 1	0	104	114	90	**103**	12
	10	119	151	144	**138**	17
Position 5	0	99	106	97	**101**	5
	10	98	102	105	**102**	4
**Control gel (no ATP injection)**						
	Time (h)	Zn^{2}+ (μM)			Average	Stdev
Position 1	0h	104	102	91	**99**	8
	10h	90	111	100	**100**	10

6. HPNPP injection experiments

These experiments were performed to understand the effect of the presence of ATP-templated assemblies on the response of the system to the chemical fuel HPNPP. Agarose gels (1 mg ml^{-1}, buffered at pH 7.0) containing a fixed amount of surfactant **1** (100 µM) and different concentrations of ATP (0 µM to 30 µM) were prepared in 6-well microtiter plates. A tiny volume (1 µL) of a concentrated HPNPP-stock solution (90 mM) was injected in the central position 1 of the gel and the absorbance at 405 nm was monitored with spatial resolution as a function of time.

6a. Change in the initial absorbance as a function of time (0-55 minutes)

The kinetic profiles were obtained by plotting the absorbance at 405 nm as a function of time for position 1 and position 2. The maximum rate (position 1) was calculated from the linear part of the absorbance vs time plot (t=10-55 minutes, with interval 5 minutes). The absorbance values for position 1 were converted to concentration unit using the calibration curve (Figure S2c) and used for calculation of the rate.

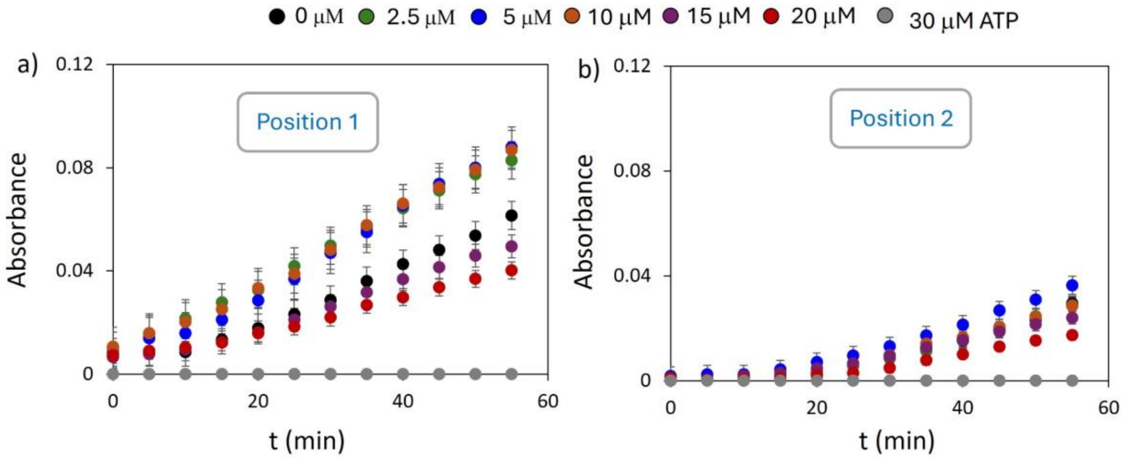

Figure S8. Absorbance at 405 nm for a) positions 1 and b) position 2 as a function of time after the injection of 1 µL of a 90 mM stock solution of HPNPP in the centre of gels containing **1** (100 µM) and ATP (0-20 µM). Experimental conditions: agarose = 1 mg/ml, [HEPES] = 5 mM, *T* = 25 °C.

6b. Longer kinetics

After the injection of a fixed amount of HPNPP (1 µL, 90 mM) in the centre of gel containing surfactant **1** (100 µM) and homogeneously distributed ATP (2.5 µM, 10 µM, 15 µM, 30 µM), the absorbance band at 405 nm was monitored in time (0h to 20h, with 0.5h interval) with spatial resolution.

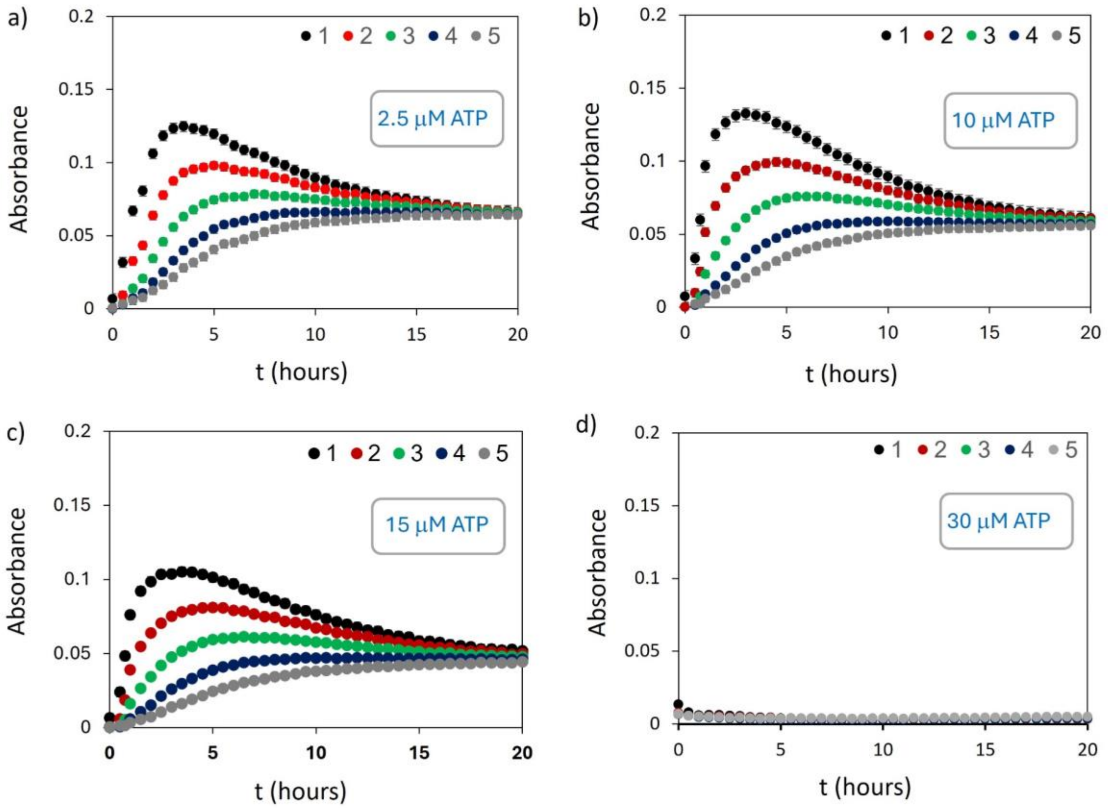

Figure S9. Absorbance at 405 nm for positions 1-5 as a function of time after the injection of 1 µL of a 90 mM stock solution of HPNPP in the position 1 containing surfactant **1** (100 µM) and different concentrations of ATP; a) 2.5 µM, b) 10.0 µM, c) 15.0 µM, d) 30.0 µM. Experimental conditions: agarose = 1 mg/ml, [HEPES] = 5 mM, *T* = 25 °C.

6c. Conversion of HPNPP

The conversion of HPNPP was calculated from the total absorbance in positions 1-7.

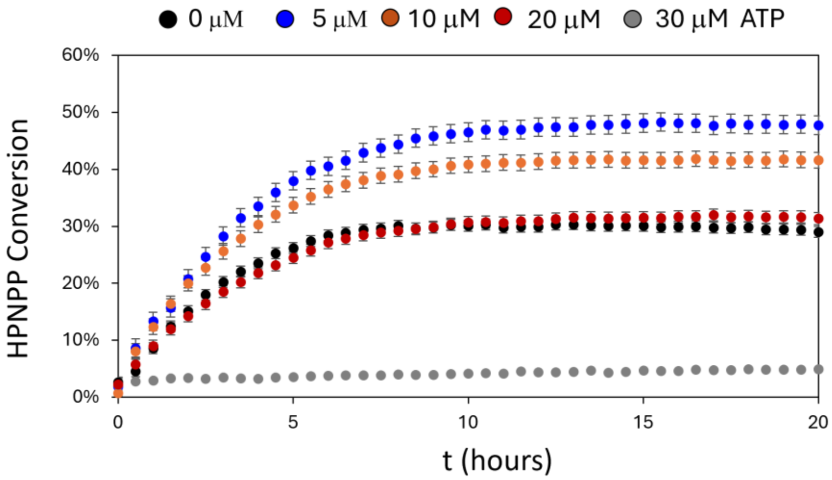

Figure S10. a) Plot of the conversion of HPNPP in the gel as a function of time for different gels. 1 μL of a 90 mM stock solution of HPNPP was injected in the centre of gel containing **1** (100 μM) and different concentrations of ATP (0-30 μM). The absorbance at 405 nm was measured for all 177 data points and by summing the absorbance values the total absorbance was calculated and converted to HPNPP conversion using calibration curves (section SI – 3). Experimental conditions: agarose = 1 mg/ml, [HEPES buffer] = 5 mM, *T* = 25 °C.

7. Repetitive injections of HPNPP

This experiment was performed to know whether the system could be reactivated by a new batch of substrate. To explore that, agarose gels (1 mg ml^{-1}, buffered at pH 7.0) containing surfactant (100 μM) and ATP (5 μM) were prepared and a tiny volume of HPNPP (1 μL, 90 mM) was injected in the position 1. The absorbance band at 405 nm was monitored in time for positions 1-5 as a function of time. After 26h, a second batch of HPNPP (1 μL, 90 mM) was injected in the same position of gel and absorbance band at 405 nm was monitored in time for positions 1-5 as a function of time. Upon injection of second batch of substrate, the increase in absorbance in position 1 increases which shows that the system can be re-activated.

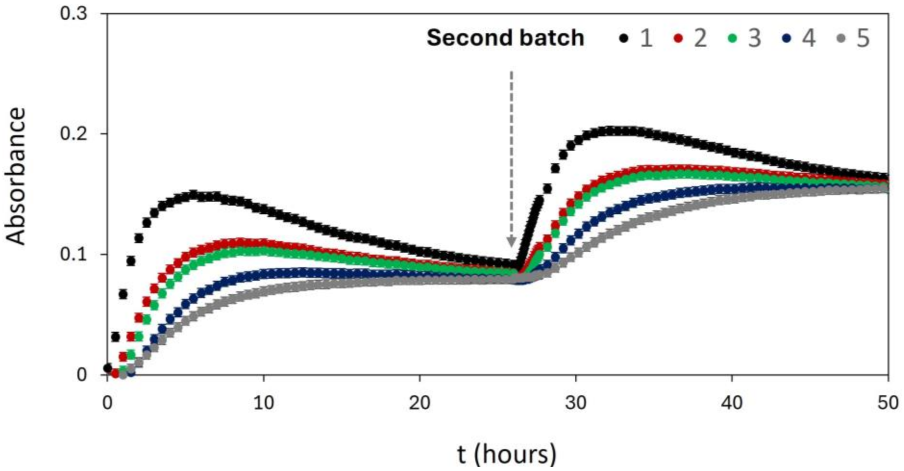

Figure S11. Absorbance at 405 nm for positions 1-5 as a function of time after the injection of 1 μL of a 90 mM stock solution of HPNPP in position 1 at t=0 h (first cycle) and t=26 h (second cycle). [**1**] = 100 μM, [ATP] = 5 μM, agarose = 1 mg/ml, [HEPES buffer] = 5 mM, *T*=25 °C.

8. Characterization of self-assembled structures by TEM (ATP injection)

In this section additional TEM images are provided of samples taken at different time points from position 1 of a gel containing **1** (100 μM) and HPNPP (125 μM) to which ATP (1 μL, 5 mM) was injected in position 1. These images complement the data reported in Figure 2.

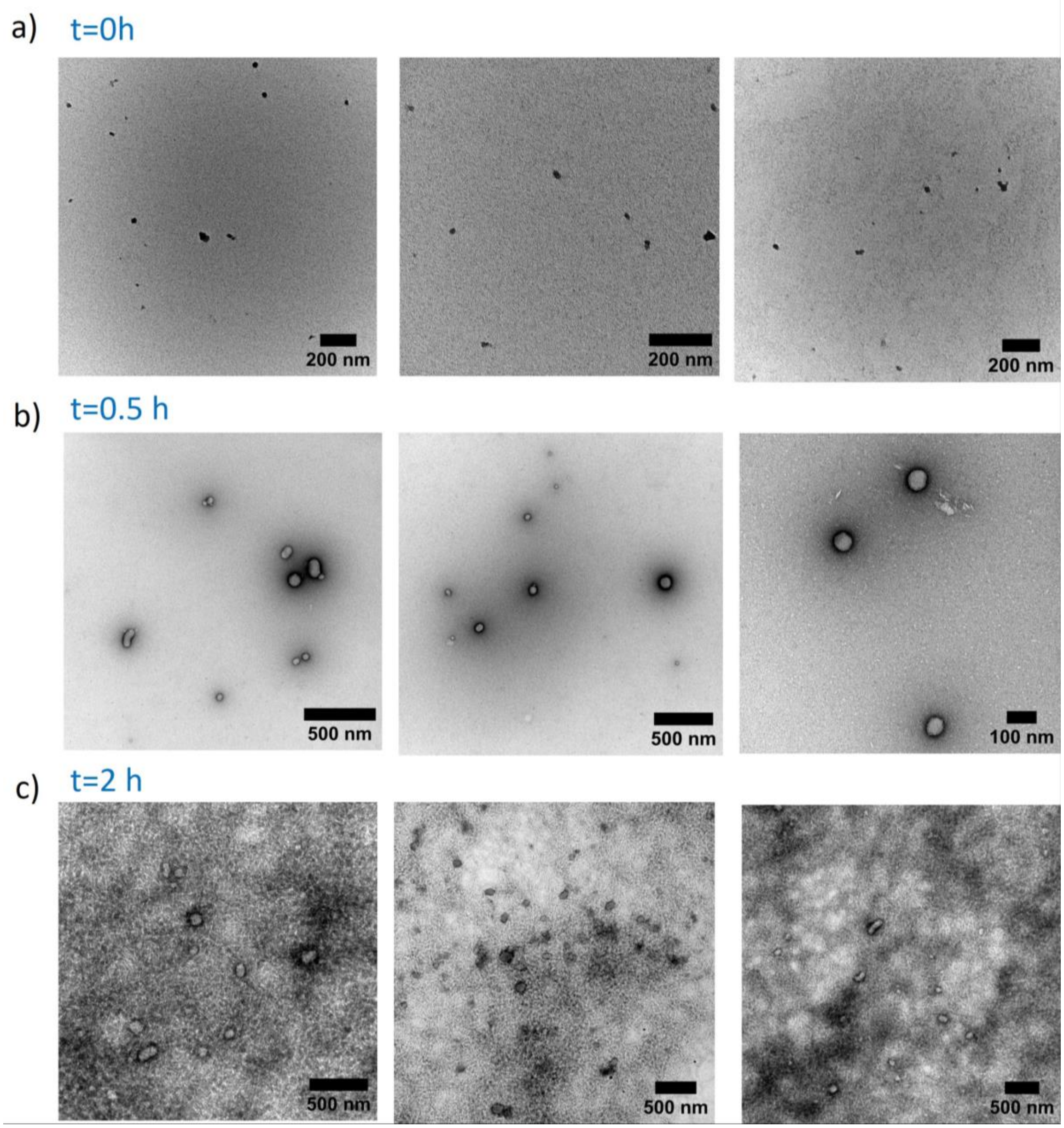

Figure S12. Additional representative transmission electron microscopy (TEM) images of position 1 of the gel taken at time = 0h (a), 0.5h (b), and 2 h (c). Experimental conditions: [**1**] = 100 μM, [HPNPP] = 125 μM, ATP (1 μL of a 5 mM stock solution) was injected in position 1. Agarose= 1 mg/ml, [HEPES]= 5 mM, T=25° C.

9. Characterization of self-assembled structures by TEM (HPNPP injection)

9a. Position 1 (5 μM ATP)

In this section additional TEM images are provided of samples taken at different time points from position 1 of a gel containing surfactant **1** (100 μM) and ATP (5 μM) to which HPNPP (1 μL of a 90 mM stock solution) was injected in position 1. These images complement the data reported in Figure 3.

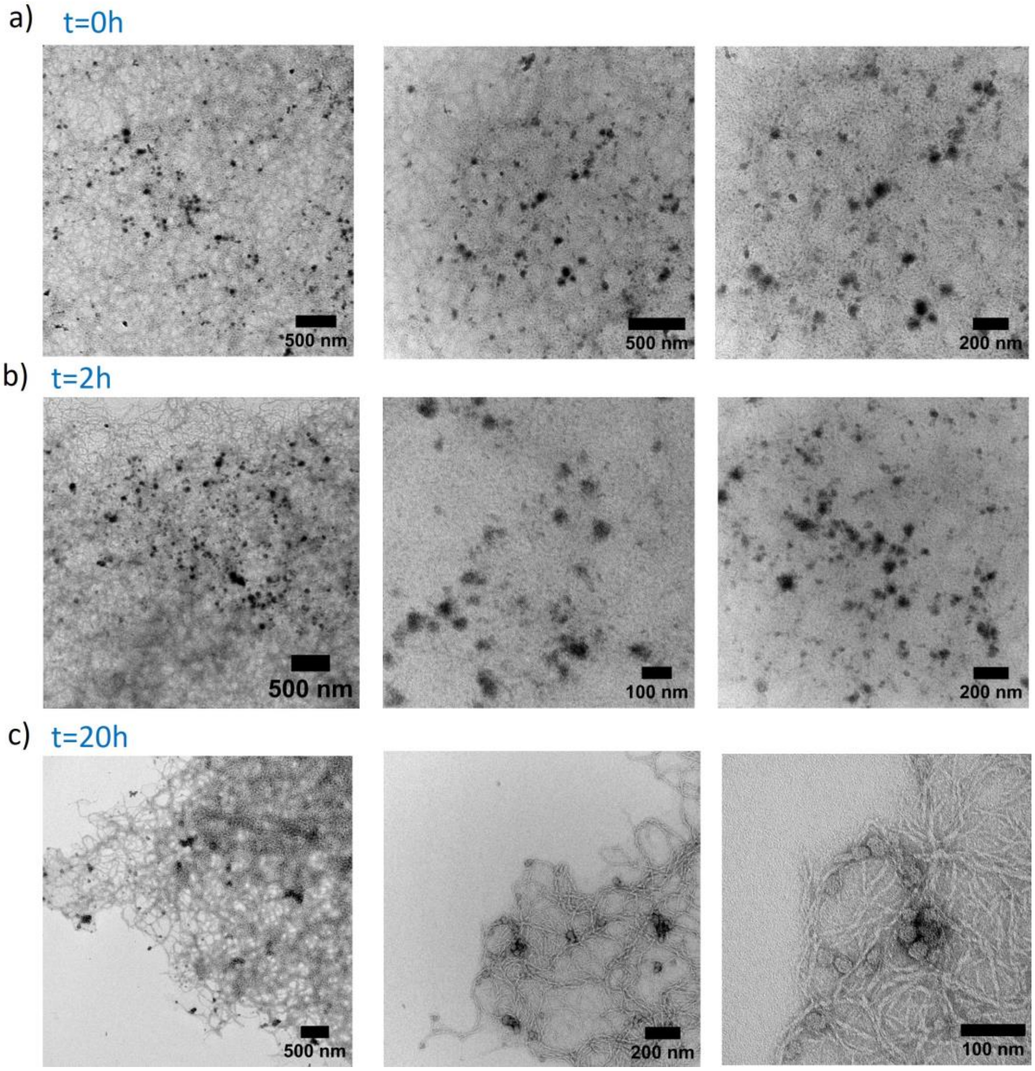

Figure S13. Additional representative transmission electron microscopy (TEM) images of position 1 of the gel taken at time = 0h (a), 2h (b), and 20 h (c). Experimental conditions: [**1**] = 100 μM, [ATP] = 5 μM. 1 μL of a 90 mM HPNPP stock solution was injected in position 1. Agarose= 1mg/ml, [HEPES]= 5 mM, T=25° C.

9b. Position 5 (5 μM ATP)

In this section additional TEM images are provided of samples taken after 2h from position 5 of a gel containing surfactant **1** (100 μM) and ATP (5 μM) to which 1 μL of a 90 mM stock solution of HPNPP was injected in position 1. These images complement the data reported in Figure 3

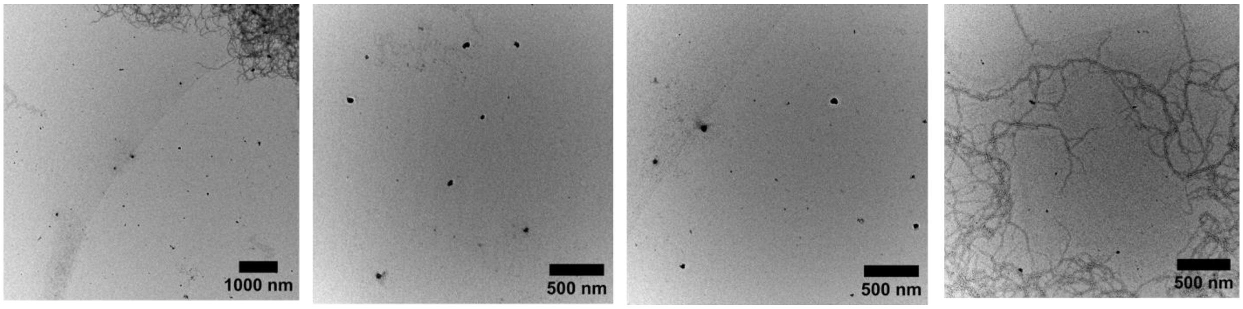

Figure S14. Transmission electron microscopy (TEM) images of position 5 of the gel taken at time 2h. Experimental conditions: [**1**] = 100 μM, [ATP] = 5 μM. 1 μL of a 90 mM HPNPP stock solution was injected in position 1. Agarose= 1 mg/ml, [HEPES]= 5 mM, T=25° C.

9c. Position 1 (20 μM ATP)

In this section additional TEM images are provided of samples taken at different time points from position 1 of a gel containing surfactant **1** (100 μM) and ATP (20 μM) to which 1 μL of a 90 mM stock solution was injected in position 1. These images complement the data reported in Figure 3.

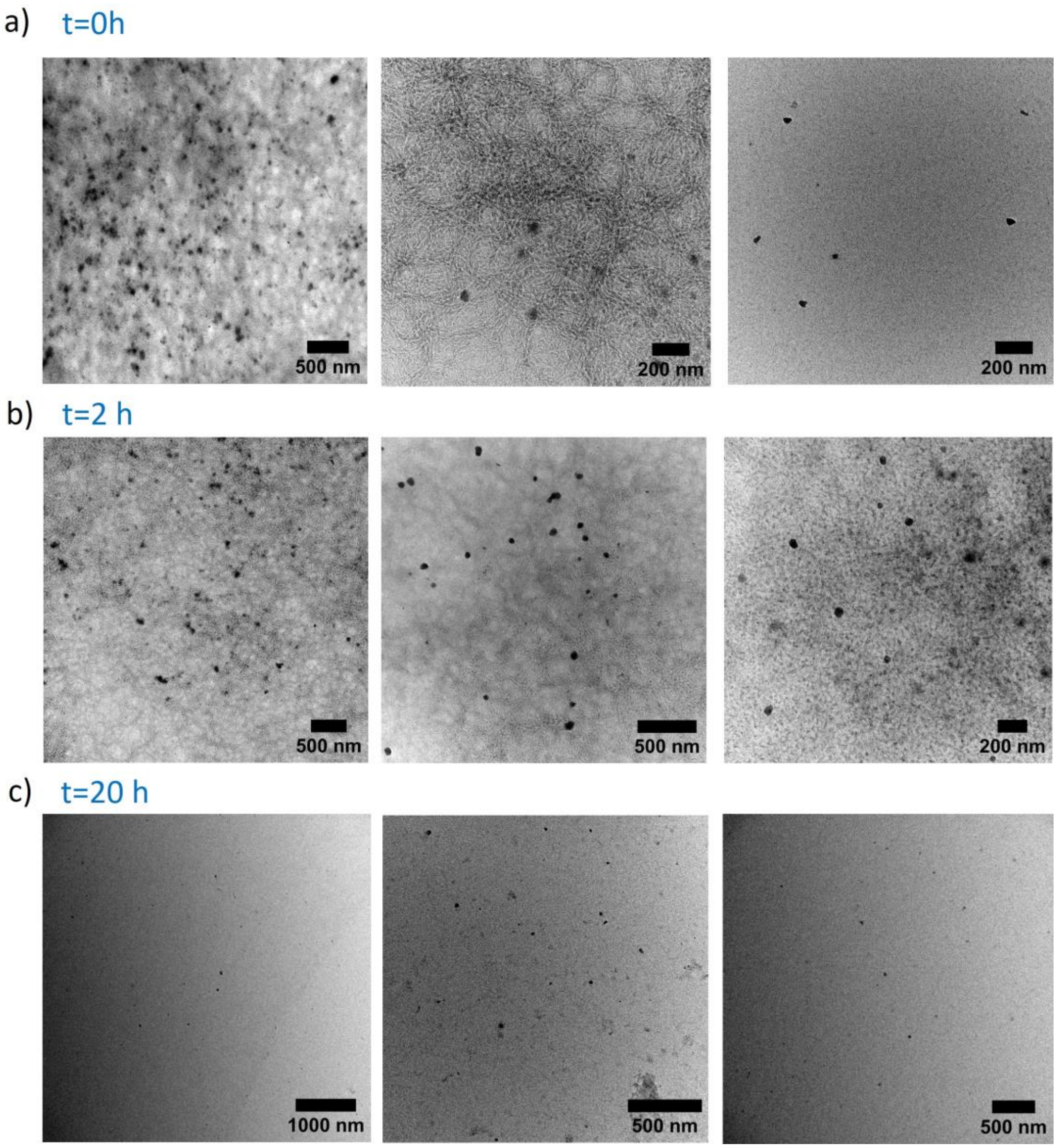

Figure S15. Additional representative transmission electron microscopy (TEM) images of position 1 of the gel taken at time = 0h (a), 2h (b), and 20 h (c). Experimental conditions: [**1**] = 100 μM, [ATP] = 20 μM. 1 μL of a 90 mM HPNPP stock solution was injected in position 1. Agarose= 1 mg/ml, [HEPES]= 5 mM, T=25° C.

10. TEM images of a gel containing homogenously distributed substrate and waste

In this section TEM images are provided of a gel in which 50/50 mixture of HPNPP and waste were homogeneously distributed.

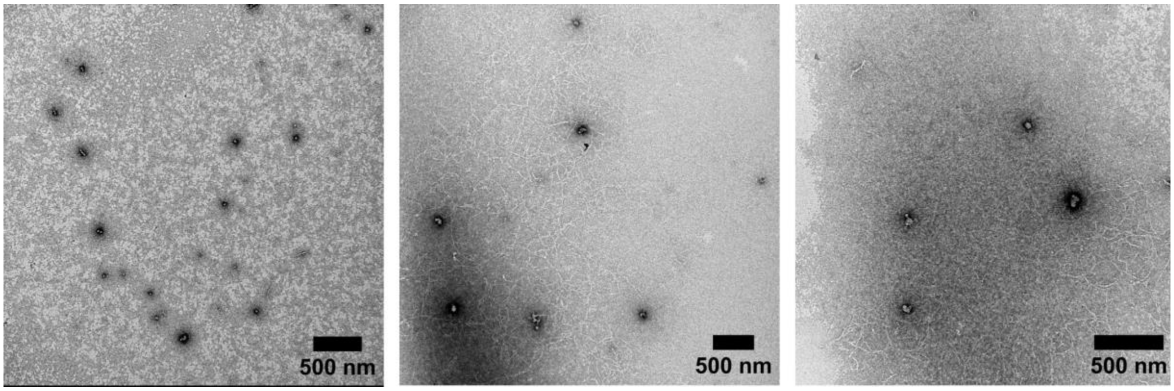

Figure S16. Transmission electron microscopy (TEM) images of sample containing [**1**] = 100 μM, [ATP] = 5 μM, [waste] = 15 μM, [HPNPP] = 15 μM. Agarose= 1mg/ml, [HEPES]= 5 mM, T=25° C.

11. Tables containing the sizes of the individual assemblies detected in the various TEM samples

Supplementary Table 3. The size of structures (nm) upon ATP-injection in position 1 of a gel containing **1** was determined from TEM images (representative images are shown in Figure 2e-g and Figure S12).

Entry	Size 0h (nm)	Size 0.5 h (nm)	Size 2.0 h (nm)
1	26	58	110
2	27	57	82
3	25	36	124
4	23	72	122
5	22	56	106
6	30	44	105
7	25	59	94
8	14	61	129
9	31	54	117
10	25	48	90
11	20	61	117
12	21	63	97
13	21	51	80
14	30	61	150
15	24	48	128
16	22	83	118
17	21	80	109
18	39	50	100
19	28	96	115
20	29	49	80
21	27	80	89
22	24	62	103
23	33	47	95
24	34	106	71
25	20	119	118
26	13	125	102
27	13	69	86
28	28	56	108
29	23	101	76
30	36	68	113
31	14	43	90
32	17	57	134
33	27	65	101
34	28	81	112
35	22	60	112
36	23		119
37	23		106
38	17		101
39	22		75
40	21		108
41	30		106
42	27		103
43	19		88
44	33		80
45	24		67
**Average**	**23**	**66**	**103**
**Stdev**	**6**	**21**	**17**

Supplementary Table 4. The size of structures (nm) in position 1 upon HPNPP injection in a gel containing **1** and ATP (5 µM) was determined from TEM images (representative images are shown in Figure 3h-j, S13)

Entry	Size 0h (nm)	Size 0.5 h (nm)	Size 2.0 h (nm)
1	50	85	41
2	50	63	39
3	43	50	47
4	46	80	75
5	50	88	58
6	50	78	25
7	42	65	31
8	62	65	26
9	71	83	51
10	76	76	25
11	53	57	32
12	34	85	35
13	29	44	30
14	38	86	30
15	46	72	50
16	60	91	30
17	63	95	35
18	43	94	41
19	60	65	56
20	42	66	36
21	37	82	40
22	65	77	35
23	65	51	55
24	56	55	28
25	54	42	34
26	67	62	44
27	59	97	39
28	67	87	51
29	51	72	46
30	65	66	39
31	73	81	31
32	62	85	36
33	42	65	31
34	65	77	58
35	46	59	34
36	47	95	72
37	61	61	60
38	42	89	25
39	50	65	22
40	57	59	30
41	53		30
42			37
43			
**Average**	**53**	**72**	**40**
**Stdev**	**11**	**14**	**12**

Supplementary Table 5. The size of structures (nm) in position 5 upon HPNPP injection of a gel containing **1** and ATP (5 µM) was determined from TEM images (representative images are shown in Figure S14).

Entry	Size 2h (nm)
1	67
2	58
3	55
4	36
5	39
6	47
7	76
8	57
9	46
10	54
11	67
12	50
13	42
14	49
15	45
16	30
17	44
18	51
19	51
20	38
21	43
22	57
23	62
24	52
25	40
26	67
**Average**	**50**
**Stdev**	**10**

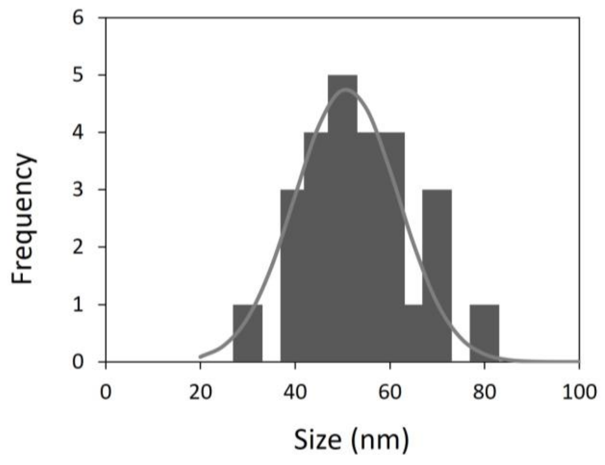

Figure S17. Size distribution of structures (Table S4) observed in TEM images (Figure S14) fitted with normal distribution function (represented with solid line).

Supplementary Table 6. The size of structures (nm) in position 1 upon HPNPP injection in a gel containing **1** and ATP (20 µM) was determined from TEM images (representative images are shown in Figure 3k-m and Figure S15)

Entry	Size 0h (nm)	Size 2h (nm)	Size 20h (nm)
1	57	57	55
2	43	39	40
3	46	50	44
4	41	43	52
5	49	57	40
6	43	51	37
7	41	42	48
8	40	44	37
9	50	38	69
10	53	47	34
11	48	53	24
12	56	56	38
13	50	51	28
14	60	49	47
15	42	52	61
16	61	63	50
17	78	52	33
18	41	40	26
19	57	73	35
20	63	52	26
21	49	53	23
22	55	47	20
23	56	58	32
24	56	66	34
25	50	50	23
26	62	49	23
27	55	58	20
28	56	45	27
29	45	48	34
30	60	44	21
31	55	45	19
32	57	50	55
33		48	
34		50	
35		55	
36		45	
37		60	
38		59	
39		54	
40		52	
41			
42			
43			
**Average**	**52**	**50**	**35**
**Stdev**	**8**	**7**	**12**

Supplementary Table 7. The size of structures (nm) in a gel in which a 1:1 mixture of HPNPP and waste (15 µM each) were homogeneously distributed was determined from TEM images (representative images are shown in Figure S16)

Entry	Size (nm)
1	45
2	26
3	43
4	35
5	29
6	36
7	38
8	59
9	51
10	59
11	34
12	45
13	42
14	35
15	40
16	50
17	44
18	31
19	36
20	31
21	36
22	29
23	28
24	47
25	50
26	33
27	41
28	41
29	38
30	36
31	32
32	43
33	50
34	32
35	46
36	44
37	53
38	34
39	32
40	28
**Average**	**39**
**Stdev**	**8**

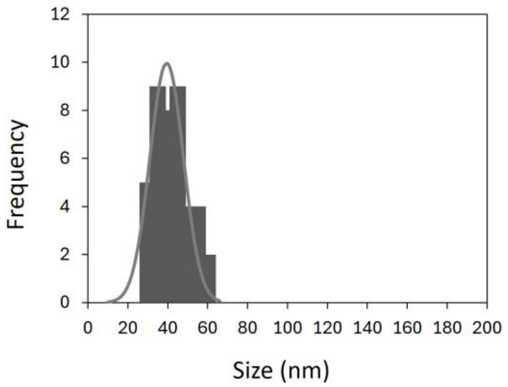

Figure S18. Size distribution of structures (table 6) observed in TEM images (Figure S16) fitted with normal distribution function (represented with solid line).

12. References

1. S. Maiti, I. Fortunati, C. Ferrante, P. Scrimin, L. J. Prins, *Nat. Chem.* **2016**, 8, 725-731.

2. D. M. Brown, D. A. Usher, *J. Chem. Soc.* **1965**, 6558-6564.

3. R. Chen, S. Neri, L.J. Prins *Nat. Nanotechnol.* **2020**, *15*, 868-874.
